# Reference gene alternatives to *Gapdh *in rodent and human heart failure gene expression studies

**DOI:** 10.1186/1471-2199-11-22

**Published:** 2010-03-23

**Authors:** Trond Brattelid, Lisbeth H Winer, Finn Olav Levy, Knut Liestøl, Ole M Sejersted, Kristin B Andersson

**Affiliations:** 1 Institute for Experimental Medical Research, Oslo University Hospital Ullevål, Oslo, Norway; 2 Department of Pharmacology, University of Oslo, Oslo, Norway; 3 Center for Heart Failure Research, Faculty of Medicine, University of Oslo, Oslo, Norway; 4 Department of Informatics, University of Oslo, Oslo, Norway; 5 Current address: National Institute of Nutrition and Seafood Research, Bergen, Norway

## Abstract

**Background:**

Quantitative real-time RT-PCR (RT-qPCR) is a highly sensitive method for mRNA quantification, but requires invariant expression of the chosen reference gene(s). In pathological myocardium, there is limited information on suitable reference genes other than the commonly used *Gapdh *mRNA and *18S *ribosomal RNA. Our aim was to evaluate and identify suitable reference genes in human failing myocardium, in rat and mouse post-myocardial infarction (post-MI) heart failure and across developmental stages in fetal and neonatal rat myocardium.

**Results:**

The abundance of *Arbp*, *Rpl32*, *Rpl4*, *Tbp*, *Polr2a*, *Hprt1*, *Pgk1*, *Ppia *and *Gapdh *mRNA and *18S *ribosomal RNA in myocardial samples was quantified by RT-qPCR. The expression variability of these transcripts was evaluated by the geNorm and Normfinder algorithms and by a variance component analysis method. Biological variability was a greater contributor to sample variability than either repeated reverse transcription or PCR reactions.

**Conclusions:**

The most stable reference genes were *Rpl32*, *Gapdh *and *Polr2a *in mouse post-infarction heart failure, *Polr2a*, *Rpl32 *and *Tbp *in rat post-infarction heart failure and *Rpl32 *and *Pgk1 *in human heart failure (ischemic disease and cardiomyopathy). The overall most stable reference genes across all three species was *Rpl32 *and *Polr2a*. In rat myocardium, all reference genes tested showed substantial variation with developmental stage, with *Rpl4 *as was most stable among the tested genes.

## Background

Quantification of mRNA abundance is a central tool in studying pathological and compensatory mechanisms in heart failure. Quantitative real-time RT-PCR (RT-qPCR) has rapidly replaced other methods, allowing quantification of many gene transcripts in limited tissue samples and more sensitive detection of weakly expressed transcripts [1]. However, this level of sensitivity requires a careful choice of method for comparison of expression data between samples. The most common strategy is to normalize the expression of a specific gene to a single reference gene, assuming that the reference gene expression is invariant between the compared physiological states. Variation in the reference gene expression between samples would therefore reflect variations in sample preparation and experimental variability. Several studies have concluded that it is difficult to identify general reference genes which can be used in all experimental settings, and that validation of the chosen reference gene(s) is important for each experimental setting [2-5].

In the heart failure literature, glyceraldehyde 3-phosphate dehydrogenase (*Gapdh*) and *18S *ribosomal RNA are the most frequently used reference genes in quantification of gene expression, see e.g. [6-8]. In rat neonatal cardiomyocytes, it was shown that *Gapdh *expression was invariant with different hypertrophic stimuli [9], but was decreased in electrically stimulated cells [10]. In a recent rat post-myocardial infarction (post-MI) heart failure study, the interpretation of changes in gene expression was dependent on the choice of *Gapdh *or *18S *as reference gene [11]. Only one recent study has examined possible reference gene candidates in human myocardium by mining public microarray data [12].

The aim of our study was to identify alternative reference genes to *Gapdh *and *18S *rRNA for use in mouse and rat post-myocardial infarction heart failure models and in human heart failure studies. Since fetal and neonatal rat cardiomyocytes are commonly used in cardiac hypertrophy and signal transduction experiments, we also evaluated a subset of candidate reference genes in neonatal and embryonic myocardium in comparison to adult myocardium. In addition we have investigated the sources of variation in gene expression quantification data in terms of RNA preparation methods, technical replicates and biological sample variability.

## Results

### Total RNA sample quality

Each total RNA sample was evaluated extensively by optical density, RNA profiling (Bioanalyzer) (Table 1) and the linearity of *18S *serial dilution curves (data not shown). The 260 nm/280 nm optical density (O.D.) ratios for rodent and human total RNA samples were similar regardless of isolation method, with no difference between the groups within each species (Table 1). The 28S/18S area ratios were consistently low (1.3), but the RNA Integrity Number (RIN) values were consistent and comparable within each species and group: Sham versus myocardial infarction (MI) or non-failing versus failing, with an overall average of 8.0.

**Table 1:** Quality evaluation of total RNA samples

			**OD 260 nm/280 nm**			**28S/18S area ratio**			**RNA integrity number (RIN)**		
		**n**	**Mean**	**SD**	**CV (%)**	**Mean**	**SD**	**CV (%)**	**Mean**	**SD**	**CV (%)**
		
Mouse	Sham	3	2.05	0.01	0.2	1.23	0.12	9.3	7.9	0.2	1.9
	MI	3	2.06	0.01	0.5	1.33	0.06	4.4	8.2	0.3	3.2
											
Rat	Sham	3	2.03	0.01	0.5	1.40	0.10	7.1	8.8	0.3	3.0
	MI	3	2.05	0.01	0.3	1.50	0.00	0.0	8.5	1.4	16.5
											
Human	Donor	4	2.03	0.03	1.3	1.36	0.36	26.5	7.2	1.9	26.0
	Failing	6	2.01	0.03	1.3	1.07	0.23	21.5	7.6	0.3	3.8

### Interspecies variation in reference gene expression

The candidate genes *Arb*, *Rpl32*, *Rpl4*, *Tbp*, *Polr2a*, *Hprt1*, *Pgk1*, *Ppia *for each species and *18S *ribosomal RNA were quantified by RT-qPCR. Due to the dominance of *18S *transcripts compared to the other reference genes of interest, the 18S data were used only as a control of the RT reaction in addition to the RNA quality control (dilution curve linearity). In mouse hearts, the post-MI/Sham ratio for *18S *was significantly higher than one (ratio 1.47, 95% CI: [1.19-1.82]). In rat hearts the *18S *post-MI/Sham ratio was also above one, but with borderline significance (ratio 1.58, [1.00-2.51]). In the human myocardium there was no significant difference (failing/non-failing ratio 1.19, [0.76-1.84]).

**Figure 1:**
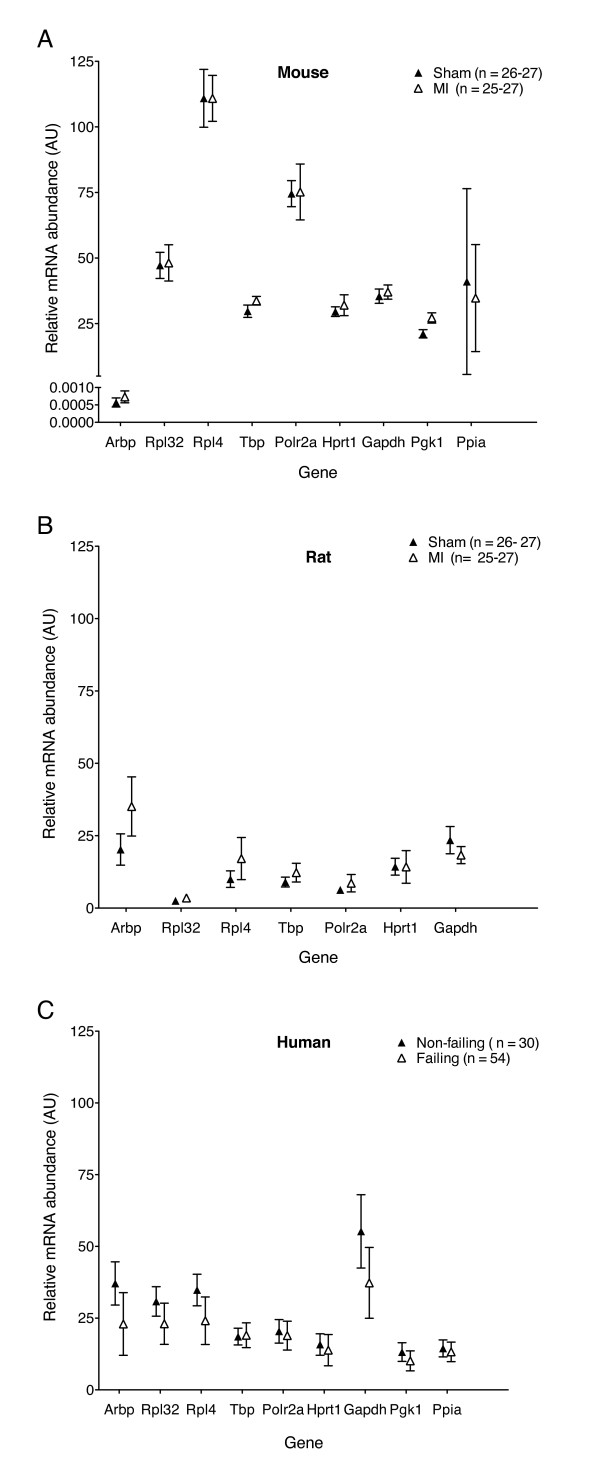
**Variation in candidate reference gene abundance in mouse, rat and human myocardium**. The indicated species-specific assays were run and Cq values converted to relative mRNA quantities (arbitrary units) using standard curves generated from species-specific pooled samples. To illustrate the total variability in the datasets for each species, the plotted data points (mean ± SD) includes all PCR runs generated from all biological samples and RT repetitions within the sham/MI or donor/failing groups for each gene. For mouse and rat groups, n = maximum of 27 PCR reactions within each group for all tested genes. For human genes, n = 30 PCR reactions were included in the donor group and 54 PCR reactions in the failing group for each gene, respectively. (A) mouse Sham and post-MI samples; (B) rat Sham and post-MI samples and (C) human non-failing (donor) and failing heart samples.

The overall variability in the relative abundance for each reference gene and sample group, MI/Sham (mouse and rat) or non-failing/failing (human) is shown in Figure 1. The mean assay coefficient of variation (CV) for each species was 8, 22 and 25% for mouse, rat and human, respectively. We next estimated the factors contributing to the gene expression variability by a variance component analysis method (see Methods). For mice, the relative size of the variance components for triplicate PCR reactions, RT repeats and biological samples were 1:1:4. The corresponding relative variance components for rat and human myocardium were 1:5:20 and 1:1:12, respectively. Overall, the biological samples, rather than sample preparation or technical replicates, contributed the most to the gene expression variability.

**Figure 2:**
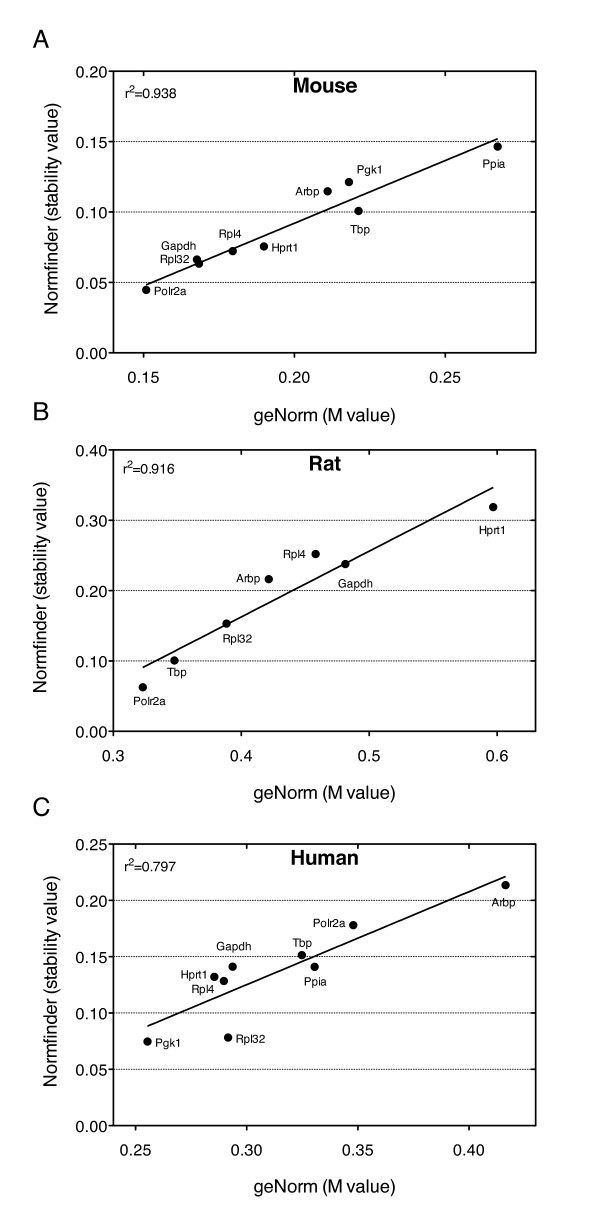
**Comparison of geNorm and Normfinder reference gene variability**. For each reference gene, the geNorm M values and Normfinder stability values were calculated separately for each RT reaction (n = 3) and averaged. The correlations between stability values by both methods are shown for (A) mouse, (B) rat and (C) human samples.

We found that gene expression variability was low for most of the analyzed reference genes. However, *Ppia *was highly variable in mice as was *Gapdh *in humans, whereas *Arbp *was moderately to highly variable in all species. In general, there was good correlation between the stability values generated by the geNorm and Normfinder algorithms for all three species (Figure 2), and by the variance component analysis (data not shown). The overall rank order of the most stable reference for each species is shown in Table 2, taking into account the results from the geNorm, Normfinder and the variance component analysis models. Stable candidate reference genes include *Rpl32*, which obtained low variability scores in all three species. The variability score for *Polr2a *was low in mouse and rat (Figure 2). However, human *Polr2a *was among the more variable genes according to both geNorm and Normfinder, while visual inspection (Figure 1C) and the variance component analysis suggested low variablity also in humans. This inconsistency may have a methodological explanation, see the discussion.

**Table 2:** The most stable reference genes in mouse, rat and human myocardium

Rank	Mouse	Rat	Human
1	Rpl32	Polr2a	Rpl32
2	Gapdh	Rpl32	Pgk1
3	Polr2a	Tbp	Ppia
4	Rpl4	Arbp	Rpl4

Reference gene rank was calculated from the combined stability results of geNorm, Normfinder and variance component analysis methods for each species.

To test the robustness of our findings, we evaluated the stability of a subset of reference genes in a second independent set of rat total RNA from Sham and post-MI myocardium (dataset 2). The samples prepared for dataset 2 differed in sample storage (dataset 1, - 80C freezer; dataset 2, RNALater), RNA isolation (dataset 1, homogenizer and Trizol; dataset 2, bead mill and Trizol) and cDNA priming methods (dataset 1, random hexamer; dataset 2, oligo-dT). Comparison of the same genes for datasets 1 and 2 yielded a nearly identical stability rank order by the geNorm method (dataset 1: *Rpl32 *≤ *Tbp *<*Arbp *<*Rpl4 *<*Gapdh; dataset 2: Tbp *<*Rpl32 *<*Arbp *<*Rpl4 *<*Gapdh*). Analysis by the Normfinder method yielded also near-identical rank orders (dataset 1: *Rpl32 *≤ *Tbp *<*Arb *<*Rpl4 *<*Gapdh *versus dataset 2: *Tbp *≤ *Rpl32 *<*Rpl4 *<*Arbp *<*Gapdh*).

### Reference gene stability across developmental stages in rat myocardium

**Figure 3:**
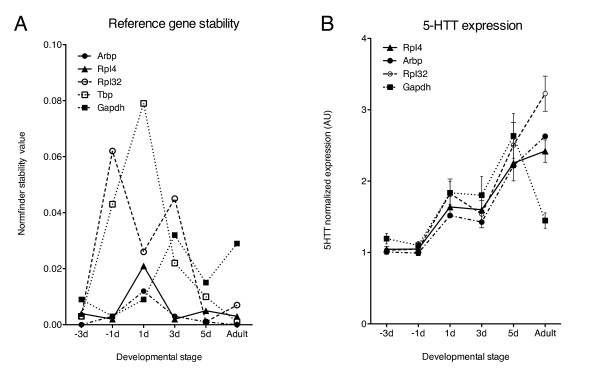
**Reference gene stability and normalization effects in fetal, neonatal and adult rat myocardium**. A) The Normfinder stability values were calculated from the relative mRNA quantities of the indicated genes in myocardial samples from rat late fetal (-3d and -1d), neonatal (1d, 3d and 5d) and adult (age 113 days) developmental stages. B) The relative abundance of the serotonin transporter *5-HTT *(*Slc6a4*) mRNA across developmental stages normalized to *Rpl4*, *Arbp*, *Rpl32 *and *Gapdh*. AU, Arbitrary units.

Fetal and neonatal cardiomyocyte preparations are used as model systems for hypertrophy responses and induction of the fetal gene program. The stability of a subset of reference genes was tested across a range of late fetal and neonatal stages and in normal *de novo *adult rat hearts. This subset was analyzed by geNorm across developmental stage (M-values in parentheses), resulting in the final ranking: *Rpl4 *(0.275), *Arbp *(0.289), *Tbp *(0.355), *Rpl32 *(0.364) and *Gapdh *(0.421). A pronounced shift in the Normfinder stability values, in particular for *Gapdh*, was observed in the transition from neonatal to adult myocardium (Figure 3A). Although none on the tested genes were very stable, Normfinder considered *Rpl4 *as the most stable reference gene across developmental stage, in agreement with the geNorm results.

The impact of normalization to a single reference gene with low (*Rpl4*, *Arbp *and *Rpl32*) or high (*Gapdh*) variability was evaluated in fetal, neonatal and adult stages (Figure 3B). Normalization of the serotonin transporter *5-HTT *(*Slc6a4*, SERT) mRNA to *Rpl4 *suggested that *5-HTT *expression increased with developmental stage. In contrast, normalization of *5-HTT *to *Gapdh *indicated only a transient increase in *5-HTT *expression.

## Discussion

We have identified several reference genes as alternatives to *Gapdh *and *18S *for use in gene expression quantification in heart failure studies. The most stable reference genes were *Rpl32*, *Gapdh *and *Polr2a *in mouse post-infarction heart failure, *Polr2a*, *Rpl32 *and *Tbp *in rat post-infarction heart failure and *Rpl32 *and *Pgk1 *in human heart failure (ischemic disease and cardiomyopathy). The overall most stable reference genes across all three species were *Rpl32 *and *Polr2a*. In rat myocardium, *Rpl4 *was the most stable reference gene across developmental stages.

It is generally accepted that the best normalization strategy would be to calculate the geometric mean of several reference genes [13]. However, this is not always feasible with limited amounts of tissue. Several of the reference gene candidates identified in this study should be sufficiently stable for single-gene normalization of gene expression data when necessary. We show that *Gapdh *is not suitable as a reference gene in human failing myocardium. A recent study using human heart failure microarray data also reached a similar conclusion [12].

Gene expression studies using fetal and neonatal heart material often use single reference gene normalization methods. Single reference gene normalization across developmental stages may be difficult because of the wide variation in expression at different developmental stages [14]. We show here a marked shift in the expression stability of several reference genes in the transition from neonatal to adult rat myocardium. This effect was particularly pronounced for *Gapdh*, which would highly influence the interpretation of results. We found that *Rpl4 *and *Rpl32 *were better reference gene candidates across developmental stages in rat myocardium.

To explore the significance of normalization to different reference genes we analyzed the expression of the serotonin 5-HT transporter (5-HTT) during fetal and neonatal development as well as in adult heart. We have previously demonstrated that the mRNA encoding the 5-HT_4 _serotonin receptor is significantly upregulated in heart failure [11,15,16] and late fetal and neonatal development (T. Brattelid, unpublished observations). Serotonin plays an important role in cardiac development [17], and the 5-HT transporter can indirectly modulate the 5-HT_4 _response by regulating the extracellular 5-HT level in cardiac muscle. Although the expression profile of 5-HTT in late fetal and neonatal heart seems to be independent of the reference gene used, the estimated 5-HTT expression in the adult heart differs significantly with the choice of reference gene (Figure 3B). *Rpl32 *and *Gapdh *represent the extreme in each direction in stability, whereas *Rpl4 *is the most stable reference gene when comparing fetal, neonatal and adult heart tissue.

In the datasets for mouse, rat dataset 1 and human reference genes, we originally included a test set of 4 commonly queried transcripts in the heart failure literature (*Apt2a2*, *Pln*, *Slc8a1*, *Nppa*) to test the impact of choice of reference genes on gene expression ratios (MI/Sham or Failing/Donor). In contrast to the fetal and neonatal rat data, the ratios and statistical significance in the adult datasets were not markedly affected by choosing between the single most stable gene, the three most stable genes, *Rpl32 *alone or *Gapdh *alone in each species (data not shown). This was due to the very small differences in the stability between the most stable reference genes in our datasets.

We found a surprising discrepancy in the variability score for *Polr2a *between rodent and human datasets by both the geNorm and Normfinder algorithms, being low in mouse and rat (Figure 2), but high in the human dataset. In contrast, the variance component analysis indicated that human *Polr2a *was quite stable, and the *Polr2a *expression levels were very similar in the donor and failing human myocardial samples (Figure 1C). We therefore examined whether this could be due to differences in the mathematical algorithms. geNorm uses pairwise comparisons of genes and assumes that the ratio between the expression values of two stable reference genes should be approximately equal in all experimental samples [13]. A gene is regarded as less stable if the pattern of variation between samples differs from the pattern observed for the majority of candidate genes, assuming that the majority are in average stable. Normfinder uses analysis of variance on log-transformed expression values, and all genes and samples are used simultaneously to estimate expected expression values [18]. A stable gene is expected to deviate only modestly from these estimates. A stability value is computed based on intra- and inter-group variations, where calculation of the latter assumes equal average expression of the genes in the groups. Thus, although in a different way from geNorm, Normfinder also assumes good "average" behavior of the test genes. In the human myocardial samples, the donor hearts tended to have higher relative quantities than failing hearts for most of the tested genes (Figure 1C). *Polr2a *expression was very similar in the donor and failing groups, thereby deviating from the majority. Therefore, both geNorm and Normfinder classify *Polr2a *as less stable. In contrast, the variance component model considers the small difference between groups as an advantage. In light of these considerations, we chose to include both human *Polr2a *and rat *Gapdh *as satisfactory reference genes. A recent report investigating gene expression variation during the estrus cycle in female rats also discussed the potential shortcomings of geNorm and Normfinder and used similar methods to the variance component model used in our study [19].

Excellent quality of total RNA is vital for obtaining reliable quantification results [20-23]. The RNA sample quality was comprehensively evaluated. Total RNA quality is traditionally estimated by the 260 nm/280 nm O.D. ratio, even though this method has several shortcomings [21]. All samples included in the study were regarded as high quality using 260 nm/280 nm O.D. ratio values. In contrast to the high *28S/18S *ratios (>2) usually obtained in cell lines, we found a low ratio (average 1.3) in myocardial tissue from all three species. Studies in human tissues have shown poor correlation between the 28S/18S ratio and RNA sample quality [20,21,23,24]. The RNA integrity number (RIN) is the most recent qualitative indicator of total RNA sample quality [20,21,24,25]. We found that the RIN values for rat and mouse myocardial total RNA (average 8.0) were consistent with values from solid tissues [20,21]. However, the RIN values for the human myocardial samples were consistently lower and the variation in the RT-qPCR data much larger than for mouse and rat, in particular for the failing hearts. This may be due to variable states of severely failing human myocardial tissue or to differences in the human sample harvesting time compared to the rapid handling of rodent myocardial samples. Even though we obtained very similar results for reference gene stability with rat datasets 1 and 2, which had considerable differences in preparation and RT priming methods, we cannot exclude that the difference in results between the human and rodent datasets may be due to differences in RNA isolation, cDNA priming methods or choice of reverse transcriptase enzymes [22,26].

In our comparison of the two independent datasets from rat Sham and post-MI myocardium, we found that the stability of the selected reference genes was independent of sample processing and cDNA priming method, thus strengthening our findings. We also found that the biological variation contributed the most to the overall quantification variability rather than technical steps such as reverse transcription and PCR amplification reactions. Thus, for myocardial samples, increasing the biological sample size rather than number of RT reaction technical replicates is a key factor for increasing the reliability of gene expression analysis.

## Conclusions

In the set of tested reference genes, the most stable reference genes were *Rpl32*, *Gapdh *and *Polr2a *in mouse post-infarction heart failure, *Polr2a*, *Rpl32 *and Tbp in rat post-infarction heart failure and *Rpl32 *and *Pgk1 *in human heart failure (ischemic disease and cardiomyopathy). The overall most stable reference genes across all three species were *Rpl32 *and *Polr2a*. In rat myocardium, *Rpl4 *was the most stable reference gene across developmental stages. These reference genes should be regarded as good *a priori *candidates, but validation of expression stability in each particular experimental setting is recommended.

## Methods

### Selection of candidate reference genes

Candidate reference genes were selected based on previous use in Northern blots and competitive RT-PCR and RT-qPCR studies in the literature: acidic ribosomal phosphoprotein P0 (*Arbp*), ribosomal protein L32 (*Rpl32*), ribosomal protein L4 (*Rpl4*), TATA-box binding protein (*Tbp*), RNA polymerase II alpha subunit (*Polr2a*), hypoxanthine guanine phosphoribosyl transferase (*Hprt1*), phosphoglycerate kinase 1 (*Pgk1*) and cyclophilin A (peptidyl prolyl isomerase A, *Ppia*), glyceraldehyde-3-phosphate dehydrogenase (*Gapdh*) and *18S *ribosomal RNA.

### Tissue material and total RNA sample sets

The B6/J mouse total RNA sample set was generated in this study. Rat and human myocardial total RNA sample sets were used previously in other studies in Wistar rats (adult dataset 1[27], adult dataset 2 [11]), fetal and neonatal rat material (Brattelid et al, in preparation), and humans [15]. Random samples that fulfilled basic "good quality" OD 260/280 ratios (approximately 2.0) were chosen from each sample set. The RNA quality of these samples was reanalyzed more extensively in this study, including RIN profiling (Table 1). Samples sizes for datasets were as follows: mouse (Sham n = 3, MI n = 3), rat dataset 1 (Sham n = 3, MI n = 3), rat dataset 2 (Sham n = 6, MI n = 6) and human (Donor n = 4, Failing n = 6).

Myocardial infarction (MI) in B6/J mice and Wistar rats was induced by ligating the left anterior descending coronary artery as described [27,28]. Age-matched Sham animals underwent the same operative procedure, but without ligating the artery. Mouse hearts were harvested after 1 week, whereas rat hearts were harvested after 6 weeks. In the original studies, criteria indicative of congestive heart failure (significantly increased left ventricular end-diastolic pressure, increased lung weight and increased lung weight/body weight ratios) were used to include mice and rats in the MI groups [28,29]. In post-MI hearts, the infarct area and border zone were removed and the remaining viable left ventricular wall and septum were harvested. In Sham and *de novo *(control) animals, the left ventricular wall and septum were harvested. Total RNA from a second set of rat left ventricles from 6-week post-MI rat hearts, age-matched Sham hearts and *de novo *animals [11], were used to test whether the combined differences in tissue storage, RNA isolation and cDNA priming methods would affect reference gene stability results (rat data set 2).

Fetal rat hearts were harvested from embryos removed under 2% isoflurane anesthesia at day 3 (n = 6) and day 1 (n = 5) before the expected term. Neonatal rat hearts were harvested at 1 (n = 10), 3 (n = 6) and 5 (n = 6) days after birth. Atria were removed and ventricles from each litter were pooled. All animals were subjected to approved protocols in accordance with the Norwegian National Committee for Animal Welfare, conforming to the *European Convention for the protection of Vertebrate animals used for Experimental and other Scientific Purposes *(Council of Europe no. 123, Strasbourg 1985). Human samples were taken from left ventricular trabeculae from non-failing donor and failing hearts. The failing hearts were from patients undergoing heart transplantation for end-stage heart failure, resulting from ischemic heart disease or dilated cardiomyopathy. Samples were obtained under ethical approval #S01025 (Oslo University Hospital Rikshospitalet, Oslo, Norway) conforming to the Declaration of Helsinki. Mouse hearts and rat hearts for dataset 1 and human samples were immediately frozen in liquid nitrogen. Adult rat hearts for dataset 2, fetal, and neonatal rat hearts were submerged and stored in RNAlater™ (Applied Biosystems/Ambion, Austin, TX, USA).

Mouse total RNA was isolated from left ventricular myocardium using a tissue homogenizer (Polytron, Kinemetica AG, Luzern, Switzerland) and SV total RNA columns as described by the manufacturer (Promega, Madison, WI, USA). Adult rat total RNA for dataset 1 was isolated from left ventricular myocardium [27] using a tissue homogenizer and the phenol/chloroform method as included in the Atlas™ Pure Total RNA Labeling kit (Clontech Laboratories Inc. Mountain View, CA, USA). Adult rat left ventricular total RNA for dataset 2, fetal and neonatal RNA samples, were obtained by washing the RNAlater-conserved tissue in sterile 0.9% NaCl, followed by homogenization in 1.5 ml Trizol by mill grinding (MM301, Retsch GmbH, Haan, Germany) with ceramic beads at 30 Hz, and isolatation of total RNA by the Trizol method [11]. Total RNA from human hearts was isolated by pulverization in liquid N_2_, homogenization with a tissue homogenizer and RNeasy columns (Qiagen Hilden, Germany) [15].: Mouse and rat dataset 1 total RNA samples were treated with DNase provided in the the RNA isolation kits, and human total RNA samples were treated with DNase I from Invitrogen (Carlsbad, CA, USA) as described by the manufacturers.

Pooled standard samples used to generate relative dilution curves for each species were prepared by mixing equal amounts of total RNA from 3 Sham and 3 MI hearts (mouse, rat), or 3 non-failing and 3 failing hearts (human), and used throughout the study.

### RNA quality control

RNA concentrations were measured (1.5 μl) in an ND-1000 spectrophotometer (NanoDrop Technologies, Wilmington, DE, USA). Samples were quality checked using RNA 6000 Nano LabChips in a 2100 Bioanalyzer (Agilent Technologies, Santa Clara, CA, USA). The RNA integrity numbers (RIN) [25] were calculated by the instrument software. *18S *serial dilution curves were run for each sample, and the linearity of the curves was used to verify the absence of inhibitory substances.

### RT-qPCR quantification

Mouse, rat (dataset 1), and human total RNA sample sets were reverse transcribed using random primers and TaqMan Reverse Transcription Reagents (P/N N808-0234, Applied Biosystems, Foster City, CA, USA). Each RNA sample was reverse transcribed independently on three separate days. All species-specific gene expression assays were run on the same cDNA generated in each independent RT reaction, thus allowing direct comparison of the relative abundance of all the reference genes within each species. The second set of *de novo*, Sham and post-MI adult rat myocardium total RNA as well as fetal and neonatal rat total RNA (5 μg) were oligo-dT-primed and transcribed with 400 U Superscript III (Invitrogen) in a 40 μl volume.

PCR assays were TaqMan^® ^Gene Expression Assays or Custom Gene Expression Assays (Applied Biosystems) (Table 3). All probes were labeled with 5'-FAM and 3'-non-fluorescent quencher. Each PCR reaction contained 12.5 μl TaqMan Universal PCR Mastermix^®^, 1.25 μl assay stock (20×), primers (900 nM), probe (250 nM) and cDNA in 25 μl final volume. PCR amplifications were run in triplicates in 96-well plates with cycling parameters 2 min 50°C; 10 min 95°C; 40 cycles of 5 s 95°C, 1 min 60°C in a 7700 (mouse) or a 7900 HT (rat, human) Sequence Detection Instrument (Applied Biosystems). Assay performance was evaluated by serial dilution curves and amplification efficiencies calculated by the formula E = 10^(1/-slope)^-1 using the slope of the relative dilution curve generated by species-specific pooled standard samples (Table 3). The specificity of each assay was verified on 2% agarose gels. To avoid loss of low copy number cDNA species, carrier tRNA was added to maintain a constant total nucleic acid input in the PCR reactions. This reduced the standard curve variability at high dilutions and in quantification of low abundance transcripts (data not shown). Quantification of *18S *and specific mRNA transcripts were run at different cDNA input levels to accommodate the large difference in 18S and mRNA abundance. For each biological sample, the baseline and threshold settings were identical for all RT runs for each gene-specific assay. Non-normalized gene expression values were calculated from species-specific standard sample serial dilution curves. The second set of control and post-MI adult rat myocardium total RNA as well as fetal and neonatal rat total RNA was analyzed using a subset of TaqMan Gene expression assays for comparison.

**Table 3:** RT-qPCR assay information

Gene Symbol	Genbank ID	Assay information	Primer location on Refseq	Amplicon (bp)	Target exons	E
**Mouse genes**						
*Rplp0 (Arbp)*	NM_007475	AB ID Mm00725448_s1		124	7	0.86
*Rpl32*	NM_172086	F: CAC CAG TCA GAC CGA TAT GTG AAA A	118-142	64	2-3	0.92
		R: TGT TGT CAA TGC CTC TGG GTT T	160-181			
		P: CCG CCA GTT TCG CTT AA	143-159			
*Rpl4*	NM_024212	F: GGT GGT TGA AGA TAA GGT TGA AGG T	500-524	71	5	0.88
		R: CTT TGA GTT TCT TGA GCA GCT GAA	547-570			
		P: CAG CCT CCT TGG TCT TC	530-546			
*Tbp*	NM_013684	AB ID Mm00446973_m1		73	4-5	0.78
*Polr2a*	NM_009089	F: CTT TGA GGA AAC GGT GGA TGT C	4293-4314	67	26	0.94
		R: TCC CTT CAT CGG GTC ACT CT	4340-4359			
		P: ATG TGC TGC TGC TTC C	4320-4335			
*Hprt1*	NM_013556	AB ID Mm00446968_m1		65	6-7	0.84
*Gapdh*	NM_008084	AB ID Mm99999915_g1		107	2-3	0.79
*Pgk1*	NM_008828	AB ID Mm00435617_m1		137	5-6	0.77
*Ppia*	NM_008907	F: GCA CTG CCA AGA CTG AAT GG	385-404	63	4-5	0.81
		R: TGC CTT CTT TCA CCT TCC CAA A	426-447			
		P: CTG GAT GGC AAG CAT G	405-420			
*Atp2a2*	NM_009722	AB ID Mm00437634_m1		59	20-21	0.87
*Pln*	NM_023129	AB ID Mm00452263_m1		71	1-2	0.83
*Slc8a1 (Ncx1)*	NM_011406	AB ID Mm00441524_m1		72	10-11	0.78
*Nppa*	NM_008725	F: GTA CAG TGC GGT GTC CAA CA	170-189		1-2	0.80
		R: CTC ATC TTC TAC CGG CAT CTT CTC	231-254			
		P: AAG AAC CTG CTA GAC CAC C	207-225			

**Rat genes**						
*Arbp (Rplp0)*	NM_022402	AB ID Rn00821065_g1		97	1-2	0.90
*Rpl32*	NM_013226	AB ID Rn00820748_g1		72	1	0.80
*Rpl4*	NM_022510	AB ID Rn00821091_g1		82	5-6	0.84
*Tbp*	NM_001004198	F: CCT CTG AGA GCT CTG GGA TTG TA	666-688	62	3-4	1.10
		R: GCC AAG ATT CAC GGT GGA TAC A	706-727			
		P: CCA CAG CTC CAA AAT A	689-704			
*Polr2a*	XM_343922	F: CGT ATC CGC ATC ATG AAC AGT GA	4181-4203	71	22-24	0.92
		R: TCA TCC ATC TTA TCC ACC ACC TCT T	4227-4251			
		P: CCT CCT CCT GCA TCT TG	4210-4226			
*Hprt1*	NM_012583	AB ID Rn01527838_g1		100	4-5	0.86
*5-HTT (Slc6a4)*	NM_013034	F: GTC ATC TGC ATC CCT ACC TAT ATC ATT	1863-1889	98	15-16	0.76
		R: GTG GGT GTT TCA GGA GTG ATA CTT T	1936-1960			
		P: AAT AAT CCG CTC CTT AAG TGT CCC CGG AGT	1905-1934			
*Gapdh*	NM_017008	AB ID Rn99999916_s1		87	3	0.93
*Atp2a2*	NM_017290	AB ID Rn00568762_m1		83	3-4	0.91
*Pln*	NM_022707	AB ID Rn01434045_m1		59	1-2	0,87
*Slc8a1 (Ncx1)*	NM_019268	AB ID Rn00570527_m1		115	5-6	0,88
*Nppa*	NM_012612	AB ID Rn00561661_m1		58	2-3	0.96

**Human genes**						
*Rplp0 (Arbp)*	NM_001002	AB ID Hs99999902_m1		105	3	0.85
*Rpl32*	NM_000994	F: CAC CAG TCA GAC CGA TAT GTC AAA A	161-185	64	2-3	0.93
		R: TGT TGT CAA TGC CTC TGG GTT T	203-224			
		P: CCG CCA GTT ACG CTT AA	186-202			
*Rpl4*	NM_000968	F: CAG CAC TGG TCA TGT CTA AAG GT	457-479	81	4-5	0.93
		R: AGC CTT CAA CTT TAT CTT CAA CTA CCA AA	537-509			
		P: CAT CGT ATT GAG GAA GTT C	480-498			
*Tbp*	NM_003194	AB ID Hs00427620_m1		91	3-4	0.97
*Polr2a*	NM_000937	AB ID Hs00172187_m1		61	1-2	0.87
*Hprt1*	NM_000194	AB ID Hs00355752_m1		N/A	N/A	0.74
*Gapdh*	NM_002046	AB ID Hs99999905_m1		122	3	0.98
*Pgk1*	NM_000291	AB ID Hs99999906_m1		75	4-5	0.74
*Ppia*	NM_021130	AB ID Hs99999904_m1		98	4	0.75
*Atp2a2*	NM_001681	AB ID Hs00544877_m1		123	5-6	0.90
*Pln*	NM_002667	AB ID Hs00160179_m1		98	1-2	0.69
*Slc8a1 (Ncx1)*	NM_021097	AB ID Hs00253432_m1		58	2-3	0.83
*Nppa*	NM_006172	AB ID Hs00383230_g1		105	1-2	0.81

18S	X03205	Eukaryotic *18S *endogenous control, part number 4319413E		187	N/A	1.06

All DNA sequences are in 5'-3' direction. F, forward primer; R, reverse primer, P, probe.

E, assay amplification efficiency; AB ID, Applied Biosystems hydrolysis probe (Taqman) Gene Expression Assay ID; Part number (Applied Biosystems). N/A, not available.

### Statistical analysis

The mean assay coefficient of variation (CV, given in %) per species was calculated by first calculating the CV of all data points for a given assay in a species, and then calculating the mean CV value for all assays within that species.

Reference gene expression variability was evaluated by geNorm [13] and Normfinder [18] analysis methods. Ratios were analyzed on the log scale to obtain approximately normally distributed observations (without outliers) and roughly equal variance across genes. Tests were two-sided, and p < 0.05 considered significant.

Mixed linear models were used to accommodate the nested experimental design and to estimate variance components and expression ratios with associated confidence intervals. Variance components included in the model were the RT reactions and the samples (nested within treatments), and with the triplicate PCR values with each run as the basic observation. We also used the sum of the variance components for the samples and the triplicates as an additional tool when evaluating gene expression stability. These types of models take into account the study design, but are limited as a general tool for evaluation of reference gene stability, because they are sensitive to large variations in total RNA quantity between samples.

## Authors' contributions

TB conceived the study, designed and performed experiments, analyzed data and drafted the manuscript. LHW participated in sample preparation, performed experiments and helped to draft the manuscript. FOL participated in study design, data analysis, provided funding and drafting of the manuscript. KL performed statistical analysis and drafted the manuscript. OMS participated in study design, provided funding and drafting of the manuscript. KBA conceived the study, designed and performed experiments, analyzed data and drafted the manuscript. All authors read and approved the final manuscript.
